# Autistic and abroad

**DOI:** 10.7554/eLife.97640

**Published:** 2024-03-15

**Authors:** Andrew K Schulz

**Affiliations:** 1 Haptic Intelligence Department, Max Planck Institute for Intelligent Systems Stuttgart Germany

**Keywords:** sparks of change, being neurodivergent in academia, autism, AuDHD, mobility, postdoc, medication

## Abstract

An AuDHD researcher recounts the highs and lows of relocating from the United States to Germany for his postdoc.

I liked to call them my mental health entourage. While navigating a stressful PhD at the Georgia Institute of Technology in Atlanta, I had a whole squad around me: my therapist and my group therapy leader, for mental health support; my nutritionist, who helped me address my disordered eating; and my psychiatrist, who prescribed my life-changing medication. Due to shame, I had spent my first few years at university trying to ‘mask’ my combined ADHD and autism spectrum disorder (also known as AuDHD). Academia was tough at times, but I had so many people in my corner helping me advance to the next day.

Then graduation came. People were hopeful and excited for me, yet deep down I was riddled with fear, doubt and conflicted emotions. I had just secured a postdoc at the Max Planck Institute for Intelligent Systems (MPI-IS) in Stuttgart, Germany. Of course, this was an amazing opportunity, but what of my mental health entourage? Would I be able to find the same support in Germany? I tried to reassure myself – after all, this was a country renowned for its almost universal healthcare. I accepted the offer and did my best to prepare for the move (Box 1).

Box 1. **How to prepare for relocating abroad.**

1. Communicate: If you feel comfortable doing so, liaise early with your future mentor/advisor and workplace to explain your needs. Ask what resources are available for medication, support etc. especially in a language you speak.
2. Coordinate your care: If you receive professional mental health support, ask your provider if they could offer remote sessions until you find someone locally. If you are on medication, have your psychiatrist share all the documentation/prescriptions you need for the maximum amount of time allowed. As soon as you can, get on the waiting lists to access mental health professionals in the area you are moving to.
3. Community: Wherever you go, finding people you can connect with is really important. Try to meet other neurodivergent individuals in your community. Seek out people who can empathize, understand and listen to your struggles, and who can offer helpful suggestions for getting through the day.
4. Check-ins: Frequent check-ins with your advisor, boss, friends, mentors and colleagues are essential. These connections can help you recalibrate and give you an opportunity to discuss the support you may need.

When I left for Stuttgart in the fall of 2022, my psychiatrist had prescribed me enough medication for 90 days, the maximum amount I was allowed to bring with me. However, getting this treatment through the German system proved to be more difficult than I expected, and for the past eighteen months I’ve had to go without the drug that allows me to cope with intense episodes of sensory overload. Without it, I often have days where I can’t live; I just exist. Rooms with too much light fill my ears with a white noise that overshadows any auditory inputs, construction sounds blur my vision, bad textures make my stomach crawl and my skin itch. Combine this with ADHD, and a single noise can trigger a downwards spiral.

Like many drugs, this medication is regulated differently in Germany and the United States. I needed to be re-assessed by a local psychiatrist before I could get a new prescription, but waiting times are long, especially for non-native speakers (it’s currently over 12 months in my region). In the end, I had my appointment just a few weeks ago; I can finally get my treatment again! That is, if I take a five-hour roundtrip to see a specific doctor, pay out of pocket, and fill out the prescription at the pharmacy on an American military base. Even then, I won’t be able to access the correct doses. I’m now back to square one and exploring what my options may be.

Adequate mental health support has also been difficult to find. One of the first things I did after relocating was to organize a meeting with a local coach… who then recommended that I manage my ADHD simply by changing my diet and getting better sleep. I feel lucky that my former therapist has agreed to meet me remotely for the time being.

In the end, my German mental health entourage looks very different from my American one. Instead of professional help, I have found a few friends, peers and mentors who have been incredibly supportive. Establishing a new social circle had felt daunting, but I’ve learned that we neurodivergent folks often find each other. Among them, I feel seen, understood and accepted. As an early-career academic, support from my supervisor has also made a real difference. Only a few months ago, I emailed them to apologize for one of my ‘just existing’ days; they came to my office, asked how they could help, and said that I never needed to apologize for this. I’ve not been living to my fullest, and of course my new squad cannot fully compensate for the challenges created by society and the healthcare system. Yet their support has allowed me to carry on, particularly in a research environment which I’ve found more isolating.

Back home, I believe that my research community was generally more aware of issues related to mental health and neurodiversity. Challenges and hurtful comments tended to emerge at the micro scale, in day-to-day interactions or incidents that people find difficult to understand or relate to, and which can lead to misunderstandings. In Germany, I feel that the stigma is around mental health, not just neurodivergence; people have questioned why I haven’t ‘graduated therapy’ yet, or why I’d even need it as a postdoc. In my experience, microaggressions are more likely to stem from a lack of knowledge. After disclosing my diagnoses, someone in the United States told me “This is great! You will finally get to check the box that says you have a disability when applying for grants!”; in my German institute, I’ve been asked “Andrew, how come your ADHD has not been cured yet?”.

I know it can be hard for others to understand what I am going through and how to support me, so I try to be honest and transparent about my experiences – even though there is a risk it will lead to a painful response. When I taught at Georgia Tech, I included a mental health statement on my syllabus to explain my struggles and meet my students and peers halfway. I’m open with my colleagues about why it takes away too much of my concentration to make eye contact when talking about research, why I may rock in my chair or move around, or why some of their remarks may be hurtful. But explaining certain concepts is even more difficult when the person you’re talking with has never heard of them, or when you don’t know the equivalent terms in their native tongue. My current institute is a melting pot, bringing together people from all over the world who have wildly different preconceived notions of mental health or neurodiversity. Misinterpretations or mistranslations can easily occur – more so when, like me, you struggle with understanding emotions and ‘reading between the lines’.

In the end, relocating to Germany has helped me grow as a person. I know I can come off as cold, that my feedback can sometimes be too stern or even mean. Just a few weeks ago, a colleague showed me a draft of their figure and I told them it looked ‘terrible’; I usually need to actively look at a person’s responses to see if I have been hurtful, and I failed to notice in the moment how much they were affected by my comment. Now that my role frequently involves helping PhD students, I have to be even more careful with how I express and regulate my emotions, including when I’m frustrated. I hope people become more empathetic towards neurodivergent individuals, but empathy goes both ways. During graduate school I constantly read books on emotional intelligence, social engineering and pop culture to learn how to relate with my peers. These are things I’ve been working on, with support and communication from those around me.

Amid these many changes, some things have stayed the same – chief of all the love for my research, science, and math. As a scientist, I have blossomed in an institute that offers incredible resources and access to some of the top researchers in my field. Working abroad has also helped me realize that making figures and visualizations is where my strength lies. The way my brain works may lead to bad sensory overload at times, but it also allows me to see research findings play out as a beautiful array of images. Every scientist appreciates the value of a good figure, no matter where they are from or how much they know about neurodiversity.

With the second year of my postdoc coming to an end, I find myself looking at the future with a lot of hope. There have been challenges, and at 29, some cracks may have started to appear in my armor. Yet, I’ve also managed to find support, understanding and people who are willing to listen. I am proud of my neurodivergence and of the community I belong to. I’ll continue to be open about my experiences, and to advocate for better mental health and neurodiversity awareness in academic spaces. My hope is that around the world, we can work towards ensuring that neurodivergent researchers do not just exist, but rather live fully, prosper and feel welcome.

## About this article

This Sparks of Change article is part of a series of articles on being neurodivergent in academia, which includes a list of tips, resources and tools collated by neurodivergent scientists.

